# Oxidative Stress Levels in the Brain Are Determined by Post-Mortem Interval and Ante-Mortem Vitamin C State but Not Alzheimer’s Disease Status

**DOI:** 10.3390/nu10070883

**Published:** 2018-07-09

**Authors:** Jared Eckman, Shilpy Dixit, Alex Nackenoff, Matthew Schrag, Fiona E. Harrison

**Affiliations:** 1 Undergraduate Program in Biology, Vanderbilt University, Nashville, TN 37232, USA; jared.eckman@vanderbilt.edu; 2 Department of Medicine, Vanderbilt University School of Medicine, Nashville, TN 37232, USA; Shilpy.Dixit.1@vanderbilt.edu; 3 Department of Neurology, Vanderbilt University School of Medicine, Nashville, TN 37232, USA; Alex.Nackenoff@vanderbilt.edu (A.N.); Matthew.Schrag@vanderbilt.edu (M.S.)

**Keywords:** oxidative stress, vitamin C, Alzheimer’s Disease, brain, post mortem interval

## Abstract

The current study highlighted several changes in measures of oxidative stress and antioxidant status that take place in the mouse brain over the course of 24 h post-mortem. Ascorbic acid (vitamin C) and glutathione both decreased significantly in cortex in as little as 2 h and malondialdehyde levels increased. Further change from baseline was observed up to 24 h, including carbonyl and sulfhydryl formation. The greatest changes were observed in brains that began with low ascorbic acid levels (gulo^−/−^ mice) compared to wild-type or 5XFAD mice. Cortical samples from nine Alzheimer’s Disease cases and five controls were also assayed under the same conditions. Post mortem intervals ranged from 6 to 47 h and all samples had low ascorbic acid levels at time of measurement. Malondialdehyde levels were lower in Alzheimer’s Disease cases. Despite a strong positive correlation between ascorbic acid and glutathione levels, no other correlations among oxidative stress measures or post mortem interval were observed. Together the data suggest that molecular changes occurring within the first hours of death may mask differences between patient groups. Care must be taken interpreting studies in human brain tissue where ante-mortem nutrient status is not known to avoid bias or confounding of results.

## 1. Introduction

Oxidative stress has long been a major focus of research in neurodegenerative diseases and in Alzheimer’s Disease in particular [1]. Theories proposing oxidative imbalance in Alzheimer’s Disease have encompassed changes at the level of the organelle, cell and organ with mechanisms related to dietary antioxidant deficiencies, transition metal metabolism defects and/or mitochondrial failure, among others [2,3,4,5]. Nevertheless, although many key mechanisms relating to oxidative stress changes to β-amyloid and tau pathology have been identified in animal models [6,7], data from human populations have been equivocal and may be subject to key methodological challenges [8]. Some increased markers of oxidative stress have been detected through post-mortem study in Alzheimer’s Disease, Parkinson’s Disease and amyotrophic lateral sclerosis patients [1]. A large meta-analysis of data studies from human pathological specimens found evidence suggesting that malondialdehyde levels may be slightly increased in the temporal and occipital lobes of the cortex and in hippocampus of Alzheimer’s Disease patients. However, it was also observed that these analyses were strongly impacted by publication bias [8]. Other markers of oxidative damage, including 4-hydroxynonenal, 8-hydroxyguanine and protein carbonylation were unchanged. Data for antioxidant levels (including ascorbic acid, alpha-tocopherol and glutathione) were too sparse for clear conclusions to be drawn [8]. Overall, it was concluded that the evidence from this meta-analysis was insufficient to support the general theory of a major change across oxidative stress processes in Alzheimer’s Disease.

The key challenges faced in direct measures from human brain tissue of disease populations are the lack of ability to sample tissue in live patients, with cerebral spinal fluid or blood markers often being used as substitutes and significant differences in post-mortem interval (PMI) for those brains donated to research. There is broad heterogeneity in ante-mortem antioxidant status (driven by diet, environmental factors and co-morbid disease states) regardless of disease status. Heterogeneity also exists in the cognitive status of those who may not have been diagnosed with Alzheimer’s Disease but who nevertheless carry a notable pathological burden at death [9]. In newer cohorts, efforts are made to minimize PMI and standardize clinical and neuropathologic features, however, historical data does not necessarily conform to these more stringent guidelines. Significant biochemical changes including oxidative stress processes and gene transcription may also take place within even the shortest time frame of two to five hours after death [10,11]. Several measures of oxidative stress can be assessed in blood without any complications from processing time and these markers have previously been researched as a possible diagnostic tool in Alzheimer’s Disease [12]. Nevertheless, data can be equivocal and other groups have found no evidence for change in peripheral markers of F_2_-isoprostanes and F_4_-neuroprostanes in Alzheimer’s Disease patients [13]. 

To address the extent to which PMI may determine changes in oxidative stress status we studied a range of markers of antioxidant status in brain in mice under low ascorbic acid (vitamin C) dietary supplementation (gulo^−/−^ mice) and mice carrying mutations derived from familial Alzheimer’s Disease populations (5XFAD) compared to controls. Brains were removed at euthanasia but not dissected and frozen until 0, 2 or 24 h following death. We hypothesized that ante-mortem antioxidant status may be a greater predictor of post-mortem measurements of oxidative stress markers than would disease status and that longer latencies to processing and freezing would have greater effects on oxidative stress markers. This study was designed to inform interpretation and design of studies in clinical samples and to highlight which processes may be the most susceptible to post-mortem changes. This research addresses a critical issue and could have implications for the future study of oxidative stress and brain health. We included many of the measures typically used and reported in clinical research studies including ascorbic acid, total glutathione, malondialdehyde, protein carbonylation and sulfhydryl groups.

## 2. Methods

**Figure 1:**
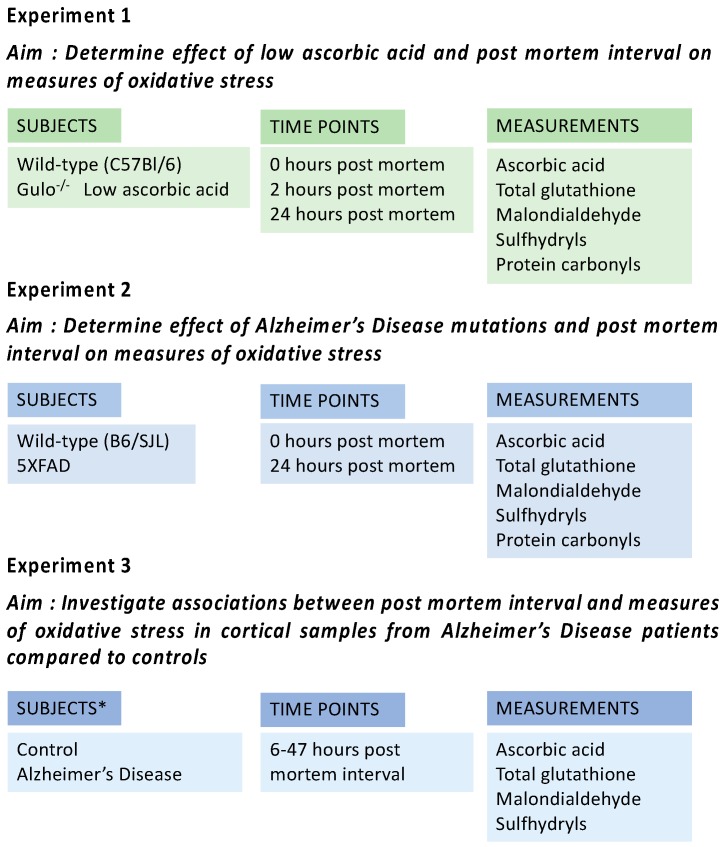
Subjects and experimental design for Experiments 1 to 3. * Please see Table 1 for details of human subjects in Experiment 3.

Overall experimental design is shown in Figure 1.

### 2.1. Animals

Gulo^−/−^ mice lack a functional copy of the enzyme l-gulonolactone oxidase and are thus unable to synthesize ascorbic acid (vitamin C). Homozygous *gulo*^−/−^ mice were originally obtained from Mutant Mouse Regional Resource Centers (http://www.mmrrc.org, MMRRC:000015-UCD) and are bred in-house and maintained on a C57BL/6J background (https://www.jax.org/strain/000664; Jackson Laboratories, Bar Harbor, ME, USA). Gulo^−/−^ mice were maintained on a low level of ascorbic acid supplementation (0.03 g/L in deionized water with 20 µL 0.5 µM EDTA per liter, made fresh twice per week) for at least 4 weeks prior to the study, which leads to brain levels of less than 30% of wild-type but does not lead to development of scurvy [14,15,16]. C57Bl6/J (wild-type) mice were used as controls. Water was available *ad libitum* and intake was not monitored.

The 5XFAD mice serve as a model for Alzheimer’s Disease and the physiological changes that result from the accumulation of amyloid protein. These mice express 5 different mutations (APP KM670/671NL, APP 1716V, APP V7171, PSEN1 M146L, PSEN1 L286V) derived from human familial Alzheimer’s Disease patients [17]. 5XFAD mice were originally obtained from Jackson labs (now MMRRC stock# 34840/34848 on a B6SJL F1 genetic background) and are maintained as a hemizygous colony through in-house breeding. Wild-type littermates were used as controls.

Experiment 1: Controls—1 male and 4 females were included at each time point, Gulo^−/−^ mice—3 males and 4 females were included in the 0 h time point, 2 males and 3 females in the 2 h time point and 3 males and 1 female in the 24 h time point. Experiment 2: Controls—4 males and 2 females were included at each time point, 5XFAD—4 males and 3 females were included at each time point. Mice were all 3–6 months old at euthanasia. All animal experiments were performed in accordance with the local Institutional Animal Care and Use Committee (IACUC) and in accordance with the National Institutes of Health Guide for the Care and Use of Laboratory Animals.

### 2.2. Human Tissue Acquisition

Experiment 3 was conducted as an exploratory investigation into changes occurring in human brain with differing disease status and PMI. This study was approved by the institutional review board of Vanderbilt University Medical Center, IRB #180287. Approximately 60 mg of occipital cortex tissue was acquired from 9 patients with Alzheimer’s disease and 5 controls, aged (27 to 102 years), with PMI ranging from 6 to 47 h (see Table 1). Tissue was preserved at the time of autopsy, rapidly frozen over liquid nitrogen and stored at −80 °C until the time of analysis. Roughly equal proportions of white and grey matter were present. Patients with Alzheimer’s Disease were diagnosed clinically with dementia, met CERAD criteria and had Braak and Braak stage IV-VI β-amyloid pathology with varying degrees of cerebral amyloid angiopathy [11,18]. Cause of death was not available for the cases with Alzheimer’s Disease as the autopsy was limited to brain analysis. Controls were clinically non-demented at the time of their death.

### 2.3. Experimental Design

Tissue processing times of 0 h (at sacrifice), 2 h (refrigerated) and 24 h (refrigerated) were selected to approximately mirror typical times to autopsy in human populations, to address the question of how time to processing impacts the critical markers of damage (see Figure 1). Brains were either removed and dissected immediately after euthanasia, with samples frozen within approximately 2 min, or stored intact, in saline in a refrigerator (+4 °C) for either 2 h or 24 h at which point cortex was removed from each sample and frozen as above. Fresh frozen samples were all stored at −80 °C until used in oxidative stress assays. In Experiment 1, oxidative changes in gulo^−/−^ mice and wild-type controls were studied at 0, 2 and 24 h In Experiment 2, 5XFAD and littermate controls were assessed at 0 and 24 h post mortem.

### 2.4. Biochemical Analyses

Ascorbic acid was measured by ion pair HPLC with electrochemical detection [19].

Glutathione was measured via the oxidation of 5,5′-dithio-bis (2-nitrobenzoic acid) (DTNB) to form 5′-thio-2-nitrobenzoic acid (TNB) measured via spectrophotometry [20,21].

Malondialdehyde was measured as thiobarbituric acid reactive substances (TBARS) [19]. The resulting product was measured by fluorescence spectrophotometry.

Protein Carbonyls were measured spectrophotometrically via reaction with DNPH with the resulting yellow product measured by spectrophotometry [22].

Sulfhydryls were measured spectrophotometrically by reduction of DTNB to TNB by thiol groups [23,24].

### 2.5. Statistical Analyses

For Experiments 1 and 2 data were analyzed by 2-way ANOVA with independent factors of Genotype and PMI. In Experiment 1, significant main effects of PMI and interactions were followed with Bonferroni-corrected multiple comparisons. In Experiment 3, each primary dependent variable was analyzed by independent-samples *t*-test (2-tailed) according to disease classification (Alzheimer’s Disease or control). Additional correlations were performed using Pearson’s R for all human samples regardless of group. Analyses were conducted using IBM SPSS Statistics, Version 24.

## 3. Results

### 3.1. Experiment 1

**Figure 2:**
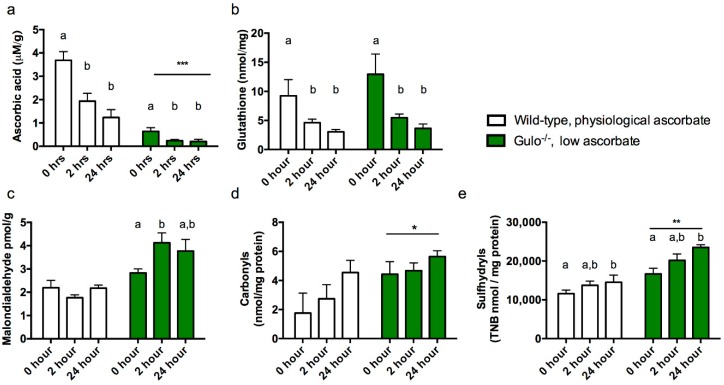
Antioxidant and oxidative stress markers in cortex of wild-type and gulo^−/−^ mice maintained at a supplement level of 0.03 g/L ascorbate in drinking water. (**a**) Ascorbic acid, (**b**) total glutathione, (**c**) malondialdehyde, (**d**) protein carbonyls and (**e**) sulfhydryl measurements from cortex at 0 h, 2 h and 24 h after sacrifice (post mortem interval, PMI). Asterisks denote main effects of ascorbate level (group) *, **, *** *p* < 0.05, 0.01, 0.001 respectively, whereas differing letters signify a main effect of PMI. Bars that do not share a letter differ from other cases within the same group at *p* < 0.05.

Ascorbic acid. We first confirmed that low ascorbic acid supplementation had appropriately lowered brain ascorbic acid in the gulo^−/−^ mice and evaluated the effect of PMI on ascorbic acid levels in all mice. As expected gulo^−/−^ mice had significantly lower brain ascorbic acid than controls (*F*_1,25_ = 33.555, *p* < 0.001) but measured ascorbic acid was also dependent on PMI with significant decreases after 24 h (*F*_2,25_ = 14.515, *p* < 0.001, Figure 2a).

Total glutathione. Since glutathione and ascorbic acid are closely related in the glutathione- ascorbic acid cycle and rodents can typically upregulate synthesis of one when the other is low [25], we also measured total glutathione levels by reduction of DTNB. Ascorbic acid level (determined by genotype) did not impact total glutathione (*F*_1,25_ = 0.811, *p* = 0.376) but glutathione did decrease with increasing PMI (*F*_2,25_ = 6.410, *p* = 0.006, Figure 2b), with the largest decrease observed within the first two hours of death (*P*s < 0.001) but significant decreases compared to 0 h at both 2 h (*p* < 0.05) and 24 h (*P*s < 0.01).

Malondialdehyde. Malondialdehyde has previously been reported to be sensitive to ascorbic acid levels in gulo^−/−^ mice [16,26] and the same effect was observed in the present study, with low ascorbic acid leading to increased malondialdehyde (*F*_1,25_ = 42.054, *p* < 0.001, Figure 2c). There was no main effect of PMI (*F*_2,25_ = 4.841, *p* = 0.197) but a significant interaction between the factors indicated that PMI affected malondialdehyde levels in gulo^−/−^ low ascorbic acid mice only (*F*_2,25_ = 4.841, *p* = 0.017) with a significant increase at 2 h PMI (*P*s < 0.01) which did not increase further at the final time point.

Carbonyls. Data was removed for one mouse (wild-type, 24 h PMI) due to very low protein levels within the sample when assayed. Protein carbonylation was greater in gulo^−/−^ mice than in controls (*F*_1,24_ = 6.039, *p* = 0.022, Figure 2d). There was no further effect of PMI (*p* > 0.131).

Sulfhydryls. Carbonyls and Sulfhydryls are measured from the same sample extract and so values for one wild-type mouse were excluded as described above. Sulfhydryl levels were significantly impacted both by ascorbic acid level (*F*_1,24_ = 34.789, *p* < 0.001) and PMI (*F*_2,24_ = 6.042, *p* = 0.007, Figure 2e) with higher levels observed in gulo^−/−^ mice and particularly by 24 h PMI compared to 0 h (*p* < 0.01).

### 3.2. Experiment 2

**Figure 3:**
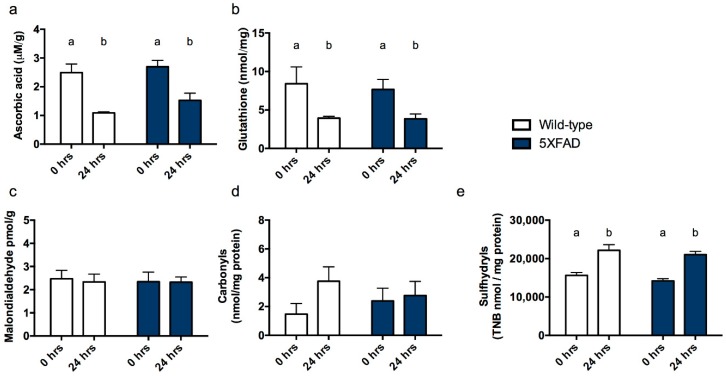
Antioxidant and oxidative stress markers in cortex of 5XFAD mice and wild-type littermate controls. (**a**) Ascorbic acid, (**b**) total glutathione, (**c**) malondialdehyde, (**d**) protein carbonyls and (**e**) sulfhydryl measurements from cortex at 0 h and 24 h following sacrifice (post mortem interval, PMI). Differing letters signify a main effect of PMI. Bars that do not share a letter differ from other cases within the same group at *p* < 0.05.

Ascorbic acid. As expected, no differences were observed according to genotype since all mice were capable of synthesizing their own ascorbate within the liver (*F*_1,23_ = 1.872, *p* = 0.184). Mirroring data from Experiment 1, ascorbic acid levels declined over the course of 24 h post-mortem (*F*_1,23_ = 29.750, *p* < 0.001, Figure 3a).

Total glutathione. Reflecting findings in ascorbic acid level and from Experiment 1, glutathione did not differ between genotypes (*F*_1,23_ = 0.039, *p* = 0.846) but did decline with time post-mortem (*F*_1,23_ = 11.084, *p* = 0.003, Figure 3b).

Malondialdehyde. Unlike in ascorbic acid deficient gulo^−/−^ mice, malondialdehyde neither varied according to genotype nor with PMI (*F*s < 0.057, *P*s > 0.814, Figure 3c).

Protein carbonyls. Protein carbonyls were also unaffected by genotype or PMI (*F*s < 0.057, *P*s > 0.814, Figure 3d).

Sulfhydryls. Sulfhydryl levels did not vary according to genotype (*F*_1,23_ = 1.863, *p* = 0.186) but did increase after 24 h storage at 4 °C in both groups (*F*_1,23_ = 50.258, *p* < 0.001, Figure 3e).

### 3.3. Experiment 3

**Figure 4:**
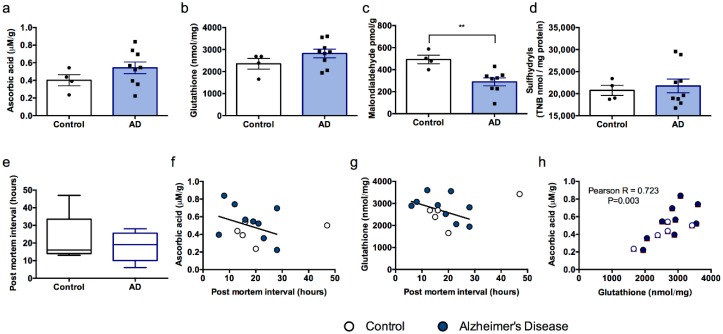
Antioxidant and oxidative stress markers do not vary according to disease or PMI in human samples. (**a**) Ascorbic acid, (**b**) total glutathione, (**c**) malondialdehyde, (**d**) sulfhydryls and (**e**) post-mortem interval (PMI) for all samples. Non-significant trends to decreasing antioxidant markers in human brain according to PMI for (**f**) ascorbic acid and (**g**) glutathione. (**h**) Ascorbic acid and glutathione levels were significantly correlated with each other. ** Control different from AD (Alzheimer’s Disease) cases, *p* < 0.01.

Human samples were processed using the same methods that were employed for mouse brain tissue. The data point corresponding to the 47 h PMI was removed from comparisons between groups since it lay greater than two standard deviations from the mean PMI for the entire data set (mean + 2S.D. = 39.99 h) and led to skewing of the data. One Alzheimer’s Disease case sample was excluded from malondialdehyde data since the value obtained was greater than two standard deviations from the group mean (mean + 2S.D. = 926.62), suggesting a technical error. Neither ascorbic acid, glutathione, nor sulfhydryls differed between control and Alzheimer’s Disease cases (ts (11) < 1.405, *P*s > 0.188, Figure 4a,b,d). However, surprisingly, malondialdehyde levels were lower in Alzheimer’s disease cases than controls (t (10) = −3.450, *p* = 0.006, Figure 4c). PMI was not different between the two groups (t (11) = 0.447, *p* = 0.664, Figure 4e).

When all subjects were included in correlational analyses (excepting the excluded malondialdehyde value) we observed no clear relationships between PMI and any of the markers of antioxidant status or oxidative damage (Table 2). A strong trend toward an inverse correlation between PMI and both ascorbic acid and glutathione was observed but was not significant in this small sample (Figure 4f,g). There was, however, a strong positive correlation between ascorbic acid and glutathione levels in these samples (Table 2, Figure 4h), further supporting the strong interaction between the two antioxidant molecules in brain tissue.

## 4. Discussion

We clearly show that ante-mortem ascorbic acid level is a critical determinant of overall oxidative stress in the gulo^−/−^ mice that, like humans, are dependent on dietary intake. In wild-type mice (and 5XFAD mice) in which synthesis can be upregulated in response to stress there is greater protection against oxidative damage. Data from Experiment 1 indicate that the greatest change in antioxidant status occurred during the first two hours following euthanasia. While malondialdehyde increased at two hours post-mortem in gulo^−/−^ mice, the same was not observed in any of the ascorbic acid-replete groups. Protein carbonylation and sulfhydryl formation also appeared to depend on ante-mortem ascorbic acid levels. The critical observation is that the change over time was different between the high and low ascorbic acid groups and, if this finding can be directly compared to human samples, it could lead to potential skewing of data according to patient nutrition status.

In this preliminary study in human cortex, there was no clear relationship between PMI and the three markers of oxidative damage (malondialdehyde, protein carbonylation and formation of sulfhydryl groups). We did not use age- and sex-matched controls for our study and no pre-study sample size prediction power analyses were conducted since the acquired data was based on the availability of samples. Nevertheless, PMI did appear to track more closely with levels of antioxidants glutathione and ascorbic acid. There is a paucity of available data on ascorbic acid levels in the human neocortex, particularly in the setting of neurodegenerative disorders, so this result is helpful to refine the interpretation of data from animal models. In comparison to the findings from the murine experiments (Figure 1 and Figure 2), human cortical ascorbic acid levels were all less than 0.8 mM which is comparable to the levels observed in ascorbic acid deficient mice at either time point and slightly lower than wild-type mice even after 24 h post-mortem. A prior study (including only 3 control and 6 Parkinson’s disease patients) found frontal lobe ascorbic acid level was 0.9 mM and unchanged with disease state [27]. Frontal lobe ascorbic acid levels are also known to decline slightly with age [28]. Ascorbic acid and glutathione were the only two factors that correlated strongly with each other in the human samples, although this is perhaps not surprising given that the values were obtained from the same extract. In contrast to expectation, malondialdehyde levels were actually lower in the Alzheimer’s Disease case brains than in the control brains but ascorbic acid, glutathione and sulfhydryls did not vary between groups. Nevertheless, without knowledge of the ante-mortem nutrient status, which was likely to be lower in the cases compared to the controls, it is difficult to interpret these findings in any meaningful way. Given that ascorbic acid depletion and deficiency (<28 and 12 uM in blood, respectively) is estimated to occur in up to 60% of aged populations, particularly those that are hospitalized [29,30,31], these findings become even more concerning. Far from representing null data, our results actually illustrate several critical points: that single measures of oxidative status are unlikely to yield accurate or useful data, that several measures of antioxidant status are sensitive to PMI and that post-mortem changes can mask ante-mortem differences. Other factors that may contribute to changes in post-mortem groups include during of agonal ante-mortem state, metabolic derangements, medication exposures and presence and degree of nutritional deficiencies [32,33]. More work in this area is merited in future to fully elucidate these processes.

The data also demonstrate that some level of Alzheimer’s Disease pathology may not significantly impact post-mortem oxidative stress. These findings do not invalidate a major body of work studying oxidative changes in Alzheimer’s Disease brains using animal and cell models, as well as serum and even brain samples from human populations. However, they strongly argue that greater consideration should be paid to the methodological challenges that may confound the data. Different dietary and environmental situations, as well as specific polymorphisms in key genes can impact oxidation-related parameters. For example, people carrying single nucleotide polymorphisms in the SVCT1 have lower circulating ascorbic acid levels due to impaired absorption in intestine and reabsorption in the kidney and are at greater risk for some health conditions including cancers [34,35,36]. Malondialdehyde is not known as a highly sensitive measure of oxidative stress although it is sensitive to changes in ascorbic acid level as shown here and in previous publications [14,16]. Measures of malondialdehyde in blood are also common in clinical studies [8]. In this study, we elected to utilize multiple individual measures of antioxidant status to illustrate differences in their response to stressors. An alternate approach not employed here would be to use a measure of overall antioxidant status or redox ability. It is important to understand the potential weaknesses of each of these measures, alone and in combination, including sensitivity to sample treatment, because they could dramatically impact interpretation of clinical data sets particularly when single measures are utilized. Further consideration of the synergistic nature of antioxidants that function as a group to maintain balance highlights the importance of the gulo^−/−^ mouse model in the study of oxidative stress and disease since, unlike humans, most other mouse and rat strains can readily maintain high levels of ascorbic acid in blood and organs through increased synthesis despite additional oxidative stressors.

Together, these data help to elucidate how oxidative stress in the brain is intensified by dietary status and post-mortem processing time. They suggest more consideration should be given to how handling of human brain tissue could be optimized in biomedical research to limit confounding factors.

## Figures and Tables

**Table 1:** Disease status, demographic details and post mortem interval for human samples used in Experiment 3.

Sample	Age	Sex	Post Mortem Interval (Hours)
Alzheimer’s Disease 1	92	Female	16
Alzheimer’s Disease 2	81	Female	21
Alzheimer’s Disease 3	63	Male	28
Alzheimer’s Disease 4	78	Female	6
Alzheimer’s Disease 5	54	Male	8
Alzheimer’s Disease 6	65	Female	19
Alzheimer’s Disease 7	64	Female	28
Alzheimer’s Disease 8	86	Male	23
Alzheimer’s Disease 9	70	Male	12
Control 1	102	Female	47
Control 2	78	Male	20
Control 3	62	Male	15
Control 4	59	Female	16
Control 5	27	Male	13

**Table 2:** 2-tailed correlations between PMI and biochemical measures in human brain tissue.

		Ascorbate	Malondialdehyde	Glutathione	Sulfhydryls	Post Mortem Interval
**Ascorbate**	Pearson Correlation		−0.123	0.768 **	0.16	−0.337
	*P* (2-tailed)		0.704	0.002	0.601	0.26
	N		12	13	13	13
**Malondialdehyde**	Pearson Correlation			-0.024	−0.303	−0.437
	*P* (2-tailed)			0.942	0.339	0.156
	N			12	12	12
**Glutathione**	Pearson Correlation				0.015	−0.432
	*P* (2-tailed)				0.962	0.141
	N				13	13
**Sulfhydryls**	Pearson Correlation					0.044
	*P* (2-tailed)					0.885
	N					13

** *p* < 0.01.
